# Exogenous Abscisic Acid Priming Modulates Water Relation Responses of Two Tomato Genotypes With Contrasting Endogenous Abscisic Acid Levels to Progressive Soil Drying Under Elevated CO_2_

**DOI:** 10.3389/fpls.2021.733658

**Published:** 2021-11-24

**Authors:** Shenglan Li, Fulai Liu

**Affiliations:** Department of Plant and Environmental Sciences, Faculty of Science, University of Copenhagen, Taastrup, Denmark

**Keywords:** elevated CO_2_, drought stress, exogenous ABA, stomatal conductance, leaf water potential

## Abstract

Plants have evolved multiple strategies to survive and adapt when confronting the changing climate, including elevated CO_2_ concentration (*e*[CO_2_]) and intensified drought stress. To explore the role of abscisic acid (ABA) in modulating the response of plant water relation characteristics to progressive drought under ambient (*a*[CO_2_], 400 ppm) and *e*[CO_2_] (800 ppm) growth environments, two tomato (*Solanum lycopersicum*) genotypes, Ailsa Craig (AC) and its ABA-deficient mutant (*flacca*), were grown in pots, treated with or without exogenous ABA, and exposed to progressive soil drying until all plant available water in the pot was depleted. The results showed that exogenous ABA application improved leaf water potential, osmotic potential, and leaf turgor and increased leaf ABA concentrations ([ABA]_leaf_) in AC and *flacca*. In both genotypes, exogenous ABA application decreased stomatal pore aperture and stomatal conductance (*g*_s_), though these effects were less pronounced in *e*[CO_2_]-grown AC and *g*_s_ of ABA-treated *flacca* was gradually increased until a soil water threshold after which *g*_s_ started to decline. In addition, ABA-treated *flacca* showed a partly restored stomatal drought response even when the accumulation of [ABA]_leaf_ was vanished, implying [ABA]_leaf_ might be not directly responsible for the decreased *g*_s_. During soil drying, [ABA]_leaf_ remained higher in *e*[CO_2_]-grown plants compared with those under *a*[CO_2_], and a high xylem sap ABA concentration was also noticed in the ABA-treated *flacca* especially under *e*[CO_2_], suggesting that *e*[CO_2_] might exert an effect on ABA degradation and/or redistribution. Collectively, a fine-tune ABA homeostasis under combined *e*[CO_2_] and drought stress allowed plants to optimize leaf gas exchange and plant water relations, yet more detailed research regarding ABA metabolism is still needed to fully explore the role of ABA in mediating plant physiological response to future drier and CO_2_-enriched climate.

## Introduction

Elevated atmospheric carbon dioxide concentrations (*e*[CO_2_]), a major component of climate change, causes an increase in global mean surface temperature (Jia et al., 2019). Along with shifting rainfall patterns and reduced freshwater resources, more frequent occurrence of drought stress has become a major constraint on plant growth and productivity (Trenberth et al., 2014; Berg and Sheffield, 2018). It is well known that both *e*[CO_2_] and drought stress influence plant water relations through the regulation of plant hormone abscisic acid (ABA), though the underlying mechanisms vary between the two environmental stimuli (Becklin et al., 2017; Li S. et al., 2020).

*e*[CO_2_] has multiple physiological effects on plant water relations. Generally, *e*[CO_2_] decreases stomatal conductance (*g*_s_), thus optimizing water use efficiency (Hatfield and Dold, 2019). Meanwhile, an improved carbon assimilation rate together with an enhanced accumulation of solutes under *e*[CO_2_] allow plants to achieve cell water homeostasis and maintain favorable leaf turgor through osmotic adjustment (OA) especially under abiotic stress (Wullschleger et al., 2002; Pérez-López et al., 2010). *e*[CO_2_]-induced positive effects on plants tend to be more pronounced under drought (Li et al., 2017; Uddin et al., 2018). In tomato and grapevine, plants grown at *e*[CO_2_] could sustain high levels of carbon assimilation rate for a longer period under drought stress due to the delayed drought effects on stomatal behavior (da Silva et al., 2017; Liu et al., 2019). Hydraulic adjustment is also one of the plants’ strategies to cope with drought stress (Comstock, 2002). Avila et al. (2020) found that coffee plants grown at *e*[CO_2_] could better maintain leaf water potential (Ψ_leaf_) and hydraulic conductance than their ambient [CO_2_]-counterparts under drought stress, thereby improving plant fitness. Although early studies have reported that *e*[CO_2_]-induced stomatal closure could reduce plant water consumption hereby increasing the availability of water in the soil during drought (Field et al., 1995), recent studies revealed that plant grown at *e*[CO_2_] might depleted soil water faster due to enlarged leaf area (Temme et al., 2018; Liu et al., 2019). Haworth et al. (2016) also indicated that the reduced effectiveness of stomatal closure at *e*[CO_2_] could impair crops’ tolerance to severe drought despite of an improved water use efficiency.

Abscisic acid is involved in both the *e*[CO_2_]- and drought stress-modulated plant water relation regulatory networks (Li S. et al., 2020). When plants are exposed to drought stress, rapid biosynthesis of ABA in roots is triggered, which is then transported from roots to leaves via the xylem to induce stomatal closure. The ABA-based root-to-shoot signaling has been considered the primary stomatal regulation mechanism in plants’ exposure to soil water deficits (Zhang et al., 1987; Zhang and Davies, 1990). However, root-sourced drought stress ABA-signaling theory has been challenged by reciprocal grafting studies on ABA biosynthetic mutants, revealing that leaf-sourced ABA could predominantly regulate stomatal aperture under rapid external pressure or long-term salinity/drought stress (Holbrook et al., 2002; Chen et al., 2003; Zhang et al., 2018). In addition, it is worthy of note that a significant proportion of root ABA was found to be derived from leaves and shoot (Ikegami et al., 2009; Ernst et al., 2010). These contrasting results raise questions about ABA homeostasis in plants when exposed to drought, including biosynthesis, catabolism, and transport. Furthermore, plants have evolved two different water management strategies: isohydric plants maintain a favorable Ψ_leaf_ and the integrity of the hydraulic system through an early stomatal closure, whereas anisohydric plants tend to keep stomata open for longer periods at a cost of hydraulic dysfunction (Sade et al., 2012). It has been reported that tomato plants exhibit an isohydric behavior, and this physiological trait is linked to the interaction of hydraulic and chemical (i.e., ABA) signals (Moshelion et al., 2015). Furthermore, our previous studies found that water management strategies of tomato plants could be modulated by CO_2_ growth environment. Under *e*[CO_2_], hydraulic signal (i.e., leaf turgor) rather than ABA predominantly controls stomatal aperture during soil drying process (Yan et al., 2017; Wei et al., 2020).

Nevertheless, ABA plays an obligatory role in various physiological responses of plants grown under the *e*[CO_2_] environment, including altered stomatal behaviors (Chater et al., 2015; Liu et al., 2019), depression on hydraulic conductance (Fang et al., 2019), and subsequently reduced water loss and enhanced leaf turgor (Huang et al., 2019; Wei et al., 2020). Many reports have addressed the role of ABA in amplifying the [CO_2_] effects on stomatal behavior (McAdam et al., 2011; Engineer et al., 2016; Hsu et al., 2018), and increases in ABA concentration in plants grown under *e*[CO_2_] have been observed in different species (Zou et al., 2007; Li B. et al., 2020). Furthermore, altered sensitivity of stomata to ABA at *e*[CO_2_] has been proposed (Rasehke, 1975; Buncec, 1998). Recently, Li B. et al. (2020) found that increased ABA content at *e*[CO_2_] could enhance soybean tolerance to drought stress, consistent with the findings by Gray et al. (2016) that *e*[CO_2_]-grown soybean represented a stronger response to ABA under soil drying. However, these results did not show any difference in hydraulic conductance, xylem pH and Ψ_leaf_ caused by *e*[CO_2_]. In tomato plants, our previous studies showed that plants grown at *e*[CO_2_] possessed a reduced sensitivity of stomata to leaf/xylem sap ABA during soil drying (Yan et al., 2017; Liu et al., 2019). A high xylem pH and a low hydraulic conductance had been reported in tomato plants grown under *e*[CO_2_] (Fang et al., 2019), which would modulate the ABA signaling thus affecting the *g*_s_ sensitivity to drought stress.

The ABA-deficit mutant *flacca* has been widely used to investigate the function of ABA due to its significantly lower ABA content compared to its wild type counterpart, Ailsa Craig (AC) (Sagi et al., 2002). Due to the higher stomatal density and greater stomatal size in *flacca* compared to the wild type under either well-watered or drought-stressed conditions (Fang et al., 2019; Innes et al., 2021; Li and Liu, 2021), it is more vulnerable to adverse environments, including soil drought and high evaporative demand conditions. By using wild type tomato and *flacca* plants, Wei et al. (2020) and Li et al. (2021) found that *e*[CO_2_] decreased *g*_s_, retarded stomatal drought response, and reduced hydraulic conductance in an ABA-dependent pathway. Namely, these effects were absent or attenuated in *flacca* plants. Some researches about exogenous ABA application have been carried out on *flacca* as well as another ABA-deficit mutant *sitiens* (Tal et al., 1979; Sharp et al., 2000; Aroca et al., 2008), together with other treatments, to explore the abnormal phenotype caused by ABA deficit. Early studies by Tal et al. (1979) and Sharp et al. (2000) have reported that the retarded plant growth and abnormal stomatal behavior in *flacca* was associated with overproduction of ethylene and could be recovered by exogenous ABA application. Therefore, to investigate the role of ABA in *e*[CO_2_]-modulated leaf gas exchange and plant water relation characteristics under drought stress, two tomato genotypes (AC and *flacca*) differing in the endogenous ABA concentrations were grown at two levels of [CO_2_] (400 and 800 ppm), treated with or without exogenous ABA and exposed to progressive soil drying. We hypothesized that (1) exogenous ABA priming would rescue the stomatal response of *flacca* to *e*[CO_2_] and soil drying and that (2) the effects of exogenous ABA on leaf gas exchange and water relations might influence *e*[CO_2_]-modulated stomatal drought response.

## Materials and Methods

### Plant Materials and Growth Conditions

Seeds of isogenic tomato cv. AC and its ABA-deficient tomato mutant (*flacca*) (*Solanum lycopersicum*) were provided by the Lancaster Environment Centre (Lancaster University, United Kingdom) and grown in climate-controlled greenhouses at the Faculty of Science, University of Copenhagen, Taastrup, Denmark. Due to the impairment in the oxidation of ABA-aldehyde to ABA, *flacca* had lower endogenous ABA concentrations compared to AC (Sagi et al., 2002). At the 4-leaf stage, the seedlings were transplanted to 4 L pots filled with 2.2 kg of peat material (Plugg-och Såjord-Dry matter ca.110 kg m^–3^, organic matter >95%, pH 5.5–6.5 and EC 1.5–2.5 mS cm^–1^). In total, 4 weeks after transplanting, perlite was used to cover the soil surface to minimize soil evaporation, and fertilizers as (NH_4_)_2_SO_4_ (2.6 g) and H_2_KPO_4_ (1.5 g) per pot were added together with irrigation water to each pot to avoid nutrient deficiency.

**FIGURE 1:**
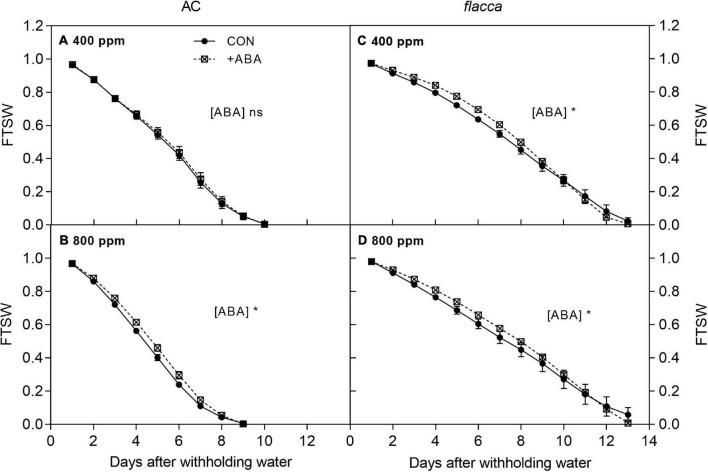
Changes in the fraction of transpirable soil water (FTSW) in AC **(A,B)** and *flacca* **(C,D)** grown under ambient (400 ppm) and elevated (800 ppm) CO_2_ concentrations during progressive soil drying. CON indicates the control treatment sprayed with water, +ABA indicates exogenous ABA application. Error bars indicate the standard error of the means (S.E.) (*N* = 3–4). *Represents the significant differences among CON and +ABA treatments in ANCOVA (Days as a covariate) at *p* < 0.05; ns denotes no significant difference.

From sowing, the plants were grown in two greenhouse cells with CO_2_ concentrations of 400 ppm (ambient CO_2_, *a*[CO_2_]) and 800 ppm (elevated CO_2_, *e*[CO_2_]), respectively. The [CO_2_] in the cells was sustained by pure CO_2_ emission from a bottle tank and distributed evenly by the internal ventilation system. The [CO_2_] in the cells was monitored every 6 s by a CO_2_ Transmitter Series GMT220 (Vaisala Group, Helsinki, Finland). The average daily [CO_2_] in each cell during treatments are shown in Figure 1. The climate conditions in the two glasshouse cells were set at: 20/16 ± 2°C day/night air temperature, 60 ± 2% relative humidity (RH), 16 h photoperiod and >300 μmol m^–2^ s^–1^ photosynthetically active radiation (PAR) supplied by sunlight plus LED lamps (Philips GreenPower LED toplighting, Denmark). The average temperature, RH, vapor pressure deficiency (VPD), and daily [CO_2_] in the cells during the experiment are shown in Supplementary Figure 1.

### Exogenous Abscisic Acid Application and Progressive Soil Drying Treatments

The sowing and growth of AC and *flacca* were conducted consecutively, and all treatments for the two genotypes were carried out separately. In each cell and genotype, 40 plants were established and constantly irrigated to 90% of the pot-holding capacity. Exogenous ABA application started 20 days after transplanting for three successive days. On each day, half of the plants were sprayed with 20 μM ABA (Sigma) twice a day on the whole plant at 10:00 and 15:00 h, respectively; the other half was sprayed with deionized water as non-ABA-treated control. All the solutions contained 0.1% (v/v) Tween-20 and 0.1% (v/v) ethanol.

In total, 1 day after finishing exogenous ABA application, four plants of each treatment (eight treatments in total) were harvested as initial control, then progressive soil drying was commenced. During soil drying, for each genotype, half of the plants were well watered to 95% of the pot’s water-holding capacity, and the remaining half was subjected to progressive soil drying by withholding irrigation from pots until all the transpirable soil water was depleted. Soil water content in the pot was expressed as the fraction of transpirable soil water (FTSW) (see below). In addition to the first harvest as initial control when FTSW was ca. 0.95, there were two destructive harvests during the soil drying period: the second was conducted when FTSW was ca. 0.50, and the last harvest was conducted at the end of soil drying when FTSW below 0.1. A total of four biological replicates (four individual plants) for each treatment were harvested. Due to the different water consumption rates between the two genotypes and the two [CO_2_] levels, AC grown at *a*[CO_2_] and *e*[CO_2_] spent 10 and 9 days to reach the end of drought stress, respectively; *flacca* grown at both [CO_2_] environments spent 13 days. Therefore, plants among different treatments were harvested on different days but at the same three FTSW points. The experimental treatments for AC and *flacca* were successively conducted, this might have caused the slightly different climatic conditions (see Supplementary Figure 1) between the two genotypes.

### Soil Water Status

Soil water status was measured daily by weighing the pots with an Analytical Balance (Sartorius Model QA35EDE-S) at 15:30 h and expressed as FTSW. The daily value of FTSW was estimated as the ratio between the amounts of transpirable soil water remaining in the pots and the total transpirable soil water (TTSW). TTSW was defined as the difference of pot weight between full water holding capacity and when all the transpirable soil water was depleted, and calculated as:


(1) TTSW=WT-fWTe

where WT_f_ is the pot weight at full water holding capacity (ca. 3.6 kg)., and WT_e_ is the pot weight at the end of soil drying (ca. 1.8 kg).

Compared to our previous study (Wei et al., 2020), here the definition of FTSW was different which resulted in the longer duration for *flacca* plants to deplete the TTSW in relation to AC plants. The reason behind this was that exogenous ABA application would affect the stomatal conductance (*g*_s_) for both genotypes, thus using a common TTSW value would allow us to compare the *g*_s_ response among the treatments on the same base. Therefore, for all treatments, the FTSW was calculated as follows:


(2) FTSW=(WT-nWT)e/TTSW

where WT_n_ is the pot weight on a given date, and WT_e_ is pot weight at the end of soil drying.

### Stomatal Pore Aperture and Stomatal Conductance Measurements

A total of 1 day after exogenous ABA application (before soil drying), stomatal pore aperture (*SA*, μm^2^) was determined on four replicates (10 stomata for one replicates) following the method described by Yan et al. (2012). Stomata were observed under a LEITZ DMRD microscope camera system (Leica Microscope and System GmbH, D 35530, Wetzlar, Germany) equipped with a digital camera. Stomatal pore aperture length (*W*_a_) and pore aperture width (*L*_a_) were measured by ImageJ software [Version 1.51k, Wayne Rasband, National Institutes of Health, United States, Java 1.6.0–24 (64 bit)]. Then *SA* was calculated as: (π × *W*_a_ × *L*_a_)/4.

During progressive soil drying, *g*_s_ (mol m^–2^ s^–1^) were measured on the last youngest upper canopy fully expanded leaves between 9:00 and 12:00 h with a portable photosynthetic system (LiCor-6400XT, LI-Cor, Lincoln, NE, United States). Measurements were performed on one leaf per plant and four biological replicates for each treatment at 22°C cuvette temperature, 1500 μmol m^–2^ s^–1^ PAR, and [CO_2_] of 400 ppm for *a*[CO_2_] and 800 ppm for *e*[CO_2_] growth environments, respectively.

### Determination of Plant Water Relations and Plant Growth

At each harvest, after gas exchange measurements, the same leaf was excised for the determination of midday leaf water potential (Ψ_leaf_, MPa) with a scholander-type pressure chamber (Soil Moisture Equipment Corp., Santa Barbara, CA, United States) following the method described by Liu et al. (2006). Then the excised leaves were cut into two parts, frozen in liquid nitrogen, and stored at −80°C for later determination of osmotic potential (Ψ_π_, MPa) and leaf ABA concentration ([ABA]_leaf_, μg g^–1^ FW). Ψ_π_ was measured at 20°C with a psychrometer (C-52 sample chambers, Wescor Inc., Logan, UT, United States) connected to a microvoltmeter (HR-33T, Wescor Inc., Logan, UT, United States). Turgor pressure (Ψ_p_, MPa) was then calculated as follows: Ψ_leaf_–Ψ_π_. Leaf dry weight (LDW) (g) and stem dry weight (SDW) (g) were determined at each harvest.

### Determination of Leaf and Xylem Sap Abscisic Acid Concentrations

At each harvest, xylem saps were collected with a scholander-type pressure chamber (AGRSCI, KVL, Denmark) according to Liu et al. (2006), then stored at −80°C for determination of xylem ABA concentrations ([ABA]_xylem_, pmol ml^–1^). [ABA]_leaf_ and [ABA]_xylem_ were determined by Enzyme-linked immunosorbent assay following the protocol of Asch (2000).

### Statistical Analyses

The responses of *g*_s_ to soil drying was described by a linear-plateau model (Faralli et al., 2019):


(3) IfFTSW>C;g=sgs⁢ini

(4) IfFTSW<C;g=sg+s⁢iniA×(FTSW-C)

where *g*_s ini_ denotes initial *g*_s_; C denotes the FTSW threshold at which y started to diverge from *g*_s ini_; and A was the slope of the linear equation.

For ABA-treated *flacca* plants, before FTSW declining to the threshold, there was a significant increasing trend of *g*_s_ during progressive soil drying. Therefore, when FTSW > C, the linear-plateau model was modified as follows:


(5) IfFTSW>C;g=sg-s⁢maxD×(C-FTSW)

where *g*_s max_ denotes maximum *g*_s_ when FTSW declined to the threshold (C), and D was the slope of the linear equation before the threshold. *g*_s max_ of non-ABA-treated *flacca* was obtained from the average of the individual replicated values when FTSW reached C. In addition, for all *flacca* plants, *g*_s end_ indicated the *g*_s_ at the end of drought treatment.

The parameters y, C, A, and D were estimated by PROC NLIN of PC SAS 9.4 (SAS Institute Inc., Cary, NC, United States, 2002–2012) and the coefficient of determination (r^2^) was calculated. Statistical comparison of each parameter obtained from the linear-plateau regression between treatments was performed by *t*-test using MedCalc statistical software 19.0.7.

Data were statistically analyzed using Microsoft Excel, SAS 9.4 (SAS Institute Inc., Cary, NC, United States, 2002–2012), SPSS 22.0 software (IBM SPSS Software, Armonk, NY, United States), and GraphPad Prism 9 software. Two-way analyses of variance (ANOVA) were performed to analyze the effects of [CO_2_] and exogenous ABA ([ABA]) on plant dry weight, *SA*, water relation variables, and ABA concentrations in AC and *flacca*. To compare the decreasing trends of FTSW during soil drying between control treatment and exogenous ABA application, the statistical differences were analyzed by analysis of covariance (ANCOVA, days after withholding water as a covariate). One-way ANOVA (Tukey’s test) and Student’s *t*-test were conducted to determine the significant differences between treatments. Principle component analysis (PCA) of *g*_s_, water relations, and leaf and xylem ABA concentrations in AC and *flacca* were performed in R version 4.0.0 (R Core Team, 2020). The relationship between *g*_s_ and [ABA]_leaf_, *g*_s_, and Ψ_p_ were evaluated by linear regression. r^2^ of the regression lines were calculated and statistical differences on the slopes and intercepts of regression lines between treatments were performed by ANCOVA.

## Results

### Plant Growth and Soil Water Depletion

In AC, exogenous ABA application did not have a significant influence on LDW and SDW, while *e*[CO_2_] increased both LDW and SDW. In *flacca*, the ABA-treated plants had higher LDW and SDW when FTSW = 0.50 and 0.05 and did not respond to *e*[CO_2_] except for an increased SDW at the last harvest (Table 1).

**TABLE 1:** Output of two-way analysis of variance (ANOVA) and means ± standard error (S.E.) (*N* = 3–4) of leaf dry weight (LDW) and stem dry weight (SDW) of AC and *flacca* grown under ambient (400 ppm) and elevated (800 ppm) CO_2_ concentrations during progressive soil drying.

**FTSW**	**[CO_2_]**	**[ABA]**	**AC**		***flacca***	
			**LDW (g)**	**SDW (g)**	**LDW (g)**	**SDW (g)**
0.95	400 ppm	CON	3.74 ± 0.24	0.89 ± 0.05	0.92 ± 0.04	0.35 ± 0.02
		+ABA	3.64 ± 0.23	0.95 ± 0.08	0.90 ± 0.11	0.33 ± 0.02
	800 ppm	CON	7.06 ± 1.22	1.85 ± 0.22	0.74 ± 0.03	0.25 ± 0.02
		+ABA	7.76 ± 0.80	2.02 ± 0.12	0.87 ± 0.02	0.25 ± 0.02
	[CO_2_]		***	***	ns	*
	[ABA]		ns	ns	ns	ns
	[CO_2_] × [ABA]		ns	ns	ns	ns
0.50	400 ppm	CON	9.52 ± 0.84	2.48 ± 0.12	2.67 ± 0.30	0.76 ± 0.05
		+ABA	10.55 ± 0.68	2.52 ± 0.10	4.15 ± 0.28	0.90 ± 0.06
	800 ppm	CON	13.36 ± 1.82	4.12 ± 0.83	2.68 ± 0.09	0.82 ± 0.03
		+ABA	12.67 ± 1.45	3.14 ± 0.35	3.74 ± 0.67	1.01 ± 0.12
	[CO_2_]		*	*	ns	ns
	[ABA]		ns	ns	*	*
	[CO_2_] × [ABA]		ns	ns	ns	ns
0.05	400 ppm	CON	14.53 ± 0.66	4.59 ± 0.23	5.01 ± 0.33	1.49 ± 0.11
		+ABA	15.79 ± 0.82	4.73 ± 0.24	7.31 ± 0.30	2.04 ± 0.06
	800 ppm	CON	18.07 ± 0.64	6.44 ± 0.43	5.04 ± 0.22	1.85 ± 0.11
		+ABA	18.15 ± 0.54	6.14 ± 0.32	7.93 ± 0.23	2.80 ± 0.17
	[CO_2_]		***	***	ns	***
	[ABA]		ns	ns	***	***
	[CO_2_] × [ABA]		ns	ns	ns	ns

*FTSW indicates the fraction of transpirable soil water. [CO_2_] indicates [CO_2_] level, [ABA] indicates without or with exogenous ABA application (expressed as CON/+ABA).*

* and *** indicate the significant differences between [CO_2_] levels or ABA treatments at *p* < 0.05, *p* < 0.001, respectively.

*ns denotes no significant difference.*

*Values are means ± standard error of the means (S.E.) (*n* = 3–4).*

After withholding irrigation, the FTSW in the pots of drought-stressed plants decreased steadily (Figure 1). In AC, only for plants grown at *e*[CO_2_], exogenous ABA slowed the soil water depletion rate during progressive soil drying. In *flacca*, at both [CO_2_] levels, exogenous ABA had a significant influence on the rates of soil water depletion. However, from 0 to 10 days, FTSW declined slower in the ABA-treated plants compared with the non-ABA-treated plants, then it declined faster on the last 3 days.

### Stomatal Pore Aperture and Stomatal Conductance

**FIGURE 2:**
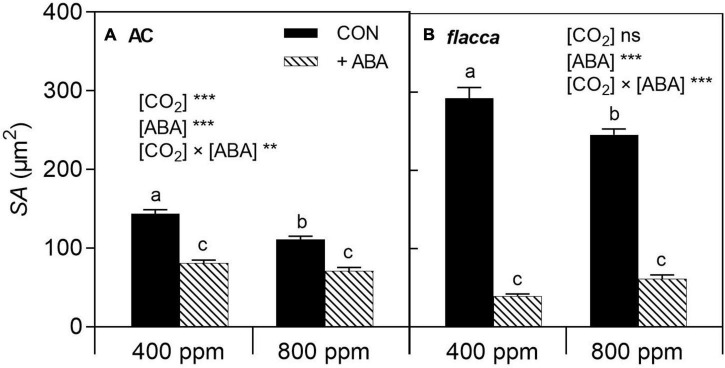
Stomatal pore aperture (*SA*) of AC **(A)** and *flacca* **(B)** grown under ambient (400 ppm) and elevated (800 ppm) CO_2_ concentrations 1 day after exogenous ABA application. CON indicates the control treatment sprayed with water, +ABA indicates exogenous ABA application. Different letters on the top of the columns indicate a significant difference between the treatments by Tukey’s test at *p* < 0.05. ** and *** indicate the significant differences between [CO_2_] and without or with exogenous ABA application ([ABA]) in two-way ANOVA at *p* < 0.01, *p* < 0.001, respectively; ns denotes no significant difference. Error bars indicate the standard error of the means (S.E.) (*N* = 40).

In both AC and *flacca*, *e*[CO_2_] decreased stomatal pore aperture (*SA*), though it was more pronounced in AC (Figure 2). One day after ABA application (before imposing soil drying), exogenous ABA decreased *SA* in both genotypes, and interactions of [CO_2_] and [ABA] were observed, indicating that the effect of *e*[CO_2_] on *SA* was eliminated by exogenous ABA application, and the depressive effect of exogenous ABA was more pronounced in *flacca* than in AC.

**FIGURE 3:**
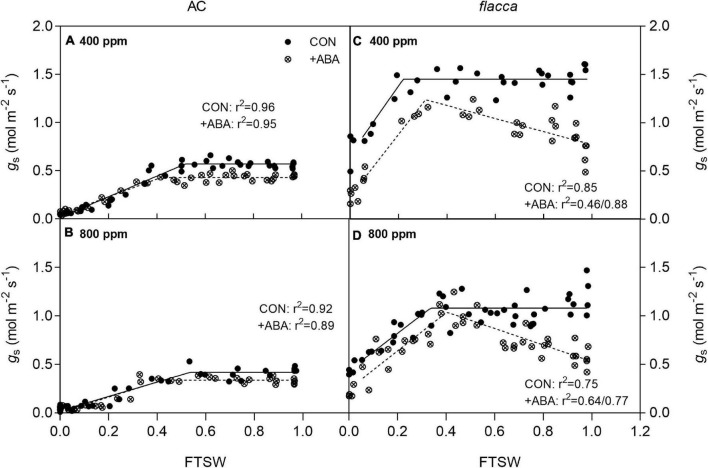
Changes of stomatal conductance (*g*_s_) in AC **(A,B)** and *flacca* **(C,D)** (total samples = 32–44, *N* = 3–4) grown under ambient (400 ppm) and elevated (800 ppm) CO_2_ concentrations during progressive soil drying. FTSW indicates the fraction of transpirable soil water. CON indicates the control treatment sprayed with water, +ABA indicates exogenous ABA application.

In AC, during progressive soil drying, plants grown at *e*[CO_2_] had significantly lower initial stomatal conductance (*g*_s ini_) compared with their *a*[CO_2_]-grown counterparts, and *g*_s_ started to decline when FTSW dropped to an FTSW threshold (C). The ABA-treated plants had constantly lower *g*_s ini_ than the non-ABA-treated pants, and C was also slightly lowered by exogenous ABA application (Figures 3A,B and Table 2).

**TABLE 2:** Output of statistical analysis of parameters derived from the linear-plateau regression of stomatal conductance (*g*_s_) of AC and *flacca* under ambient (400 ppm) and elevated (800 ppm) CO_2_ concentrations response to the reduction in the fraction of transpirable soil water (FTSW).

**[CO_2_]**	**[ABA]**	**AC**			***flacca***			
		**C**	***g*_s ini_**	***g*_s end_**	**C**	***g*_s ini_**	***g*_s end_**	***g*_s max_**
400 ppm	CON	0.52 ± 0.02A	0.57 ± 0.01aB	0.05 ± 0.01B	0.22 ± 0.03bB	1.56 ± 0.03aA	0.76 ± 0.09aA	1.45 ± 0.02a
	+ABA	0.41 ± 0.02A	0.43 ± 0.01bB	0.06 ± 0.01B	0.31 ± 0.03abB	0.66 ± 0.07cA	0.23 ± 0.03bA	1.24 ± 0.06b
800 ppm	CON	0.53 ± 0.05A	0.42 ± 0.01bB	0.05 ± 0.02B	0.34 ± 0.04abB	1.22 ± 0.10bA	0.53 ± 0.07aA	1.08 ± 0.02b
	+ABA	0.42 ± 0.04	0.34 ± 0.01cB	0.06 ± 0.01B	0.40 ± 0.03a	0.55 ± 0.05cA	0.21 ± 0.03bA	1.04 ± 0.04b

*[CO_2_] indicates [CO_2_] level, [ABA] indicates without or with exogenous ABA application (expressed as CON/+ABA). The data is presented in Figure 3. Values are means ± SE.*

*Lowercase letters indicate the significant difference among the [CO_2_] and [ABA] treatments in each column; capital letters indicate the significant difference between AC and *flacca*; no letter indicates no significant difference.*

*C, the threshold at *g*_*s*_ which started to decline due to drought stress; *g*_*s ini*_, the initial values of variables when plants were not significantly affected by drought; *g*_*s end*_, the final values of variables at the end of drought stress. For *flacca*, *g*_*s max*_ indicates the maximum values of variables before it starting to decrease.*

In *flacca*, 1 day after exogenous ABA application, depression on *g*_s ini_ ranged from 50 to 58% at *a*[CO_2_] and e[CO_2_] environment, respectively. During soil drying, there were clear increasing trends of *g*_s_ in the ABA-treated plants before FTSW reaching to the threshold, and when FTSW decreased to C, only the ABA-treated plants grown at *a*[CO_2_] still had lower maximum *g*_s_ (*g*_s max_) than the non-ABA-treated plants. Moreover, the FTSW thresholds were slightly advanced by exogenous ABA application. At the end of soil drying, exogenous ABA treatment still lowered *g*_s_ (*g*_s end_) of plants grown at both [CO_2_] levels, but all *flacca* plants possessed significantly higher *g*_s end_ than AC. In addition, *e*[CO_2_] decreased *g*_s_ in non-ABA-treated *flacca* and slightly advanced C (Figures 3C,D and Table 2).

### Plant Water Relation Characteristics

**FIGURE 4:**
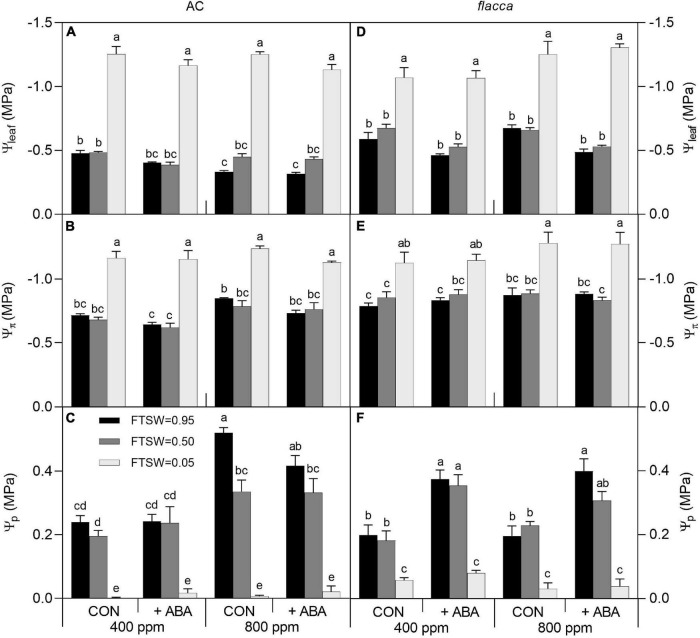
Leaf water potential (Ψ_leaf_), osmotic potential (Ψ_π_), and turgor pressure (Ψ_p_) in AC **(A–C)** and *flacca* **(D–F)** grown under ambient (400 ppm) and elevated (800 ppm) CO_2_ concentrations during progressive soil drying. CON indicates the control treatment sprayed with water, +ABA indicates exogenous ABA application. FTSW indicates the fraction of transpirable soil water. Different letters on the top of the columns indicate a significant difference between the treatments by Tukey’s test at *p* < 0.05. Error bars indicate the standard error of the means (S.E.) (*N* = 3–4).

A total of 1 day after exogenous ABA application, before imposing soil drying (i.e., FTSW = 0.95), the ABA-treated AC plants had higher leaf water potential (Ψ_leaf_) and osmotic potential (Ψ_π_), whereas the ABA-treated *flacca* plants possessed higher Ψ_leaf_ and turgor pressure (Ψ_p_). In addition, slight interactions of [CO_2_] and [ABA] were observed in Ψ_leaf_ and Ψ_p_ of AC when FTSW = 0.95, showing that the effects of exogenous ABA became less significant when plants were grown at *e*[CO_2_]. At the end of soil drying (i.e., FTSW = 0.05), all these plant water relation variables significantly decreased by drought stress, and exogenous ABA had no influence on water relations of *flacca*, whereas it still slightly increased Ψ_leaf_ in AC. Moreover, *e*[CO_2_] improved Ψ_leaf_, Ψ_π_, and Ψ_p_ of AC plants compared to *a*[CO_2_], though these effects being diminished along with soil drying (Figure 4 and Table 3).

**TABLE 3:** Results of two-way ANOVA test showing the statistical significance of the effects of [CO_2_] and without or with exogenous ABA application ([ABA]) on leaf water potential (Ψ_leaf_), osmotic potential (Ψ_π_), turgor pressure (Ψ_p_), leaf and xylem sap ABA concentrations ([ABA]_leaf_, [ABA]_xylem_) in AC and *flacca* during progressive soil drying.

**Genotype**	**FTSW**	**Factor**	**Ψ_leaf_ (MPa)**	**Ψ_π_ (MPa)**	**Ψ_p_ (MPa)**	**[ABA]_leaf_ (μg g^–1^ FW)**	**[ABA]_xylem_ (pmol ml^–1^)**
AC	0.95	[CO_2_]	***	***	***	ns	ns
		[ABA]	**	***	ns	**	ns
		[CO_2_] × [ABA]	*	ns	*	ns	ns
	0.50	[CO_2_]	ns	**	*	**	ns
		[ABA]	**	ns	ns	ns	ns
		[CO_2_] × [ABA]	ns	ns	ns	*	ns
	0.05	[CO_2_]	ns	ns	ns	ns	ns
		[ABA]	*	ns	ns	ns	ns
		[CO_2_] × [ABA]	ns	ns	ns	ns	ns
*flacca*	0.95	[CO_2_]	ns	*	ns	*	ns
		[ABA]	***	ns	***	***	**
		[CO_2_] × [ABA]	ns	ns	ns	**	ns
	0.50	[CO_2_]	ns	ns	ns	***	ns
		[ABA]	***	ns	**	***	ns
		[CO_2_] × [ABA]	ns	ns	ns	*	ns
	0.05	[CO_2_]	*	ns	ns	ns	***
		[ABA]	ns	ns	ns	ns	**
		[CO_2_] × [ABA]	ns	ns	ns	ns	***

*FTSW indicates the fraction of transpirable soil water.*

*, **, and *** indicate the significant differences between treatments at *p* < 0.05, *p* < 0.01, *p* < 0.001, respectively.

*ns denotes no significant difference.*

**FIGURE 5:**
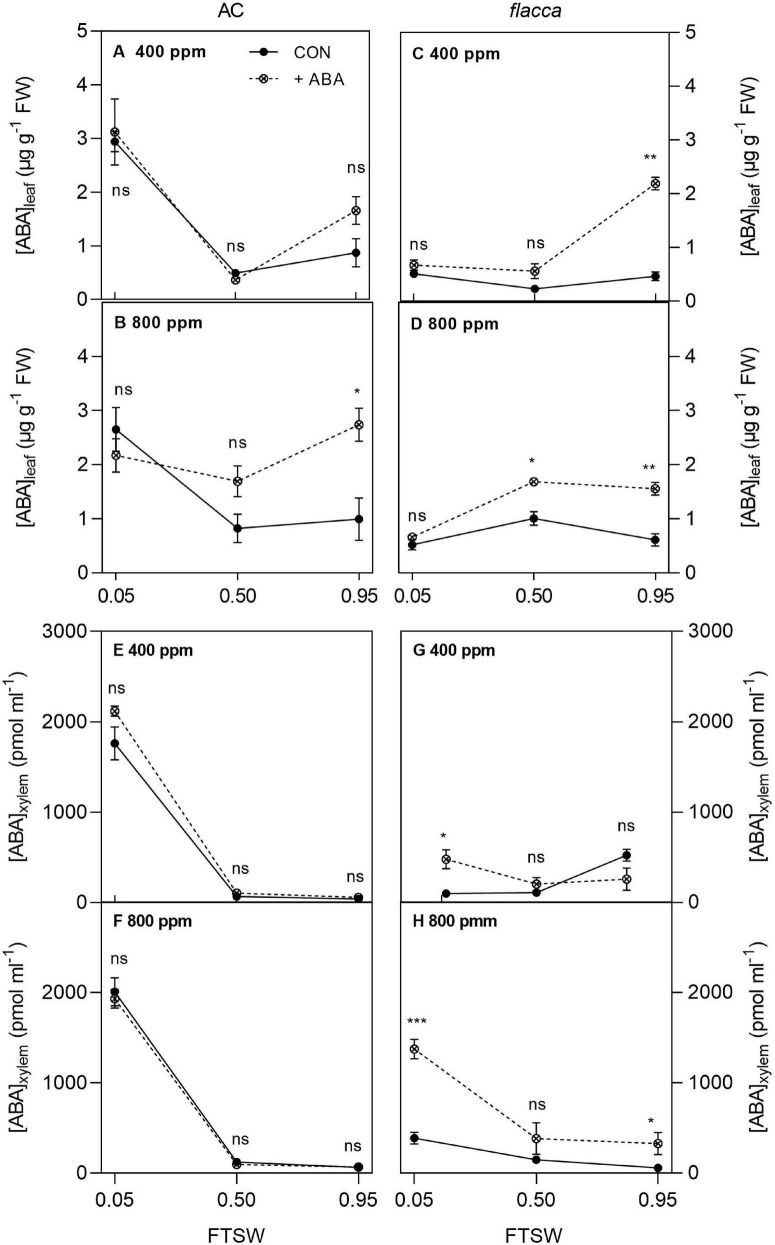
Leaf and xylem sap ABA concentrations ([ABA]_leaf_, **A–D**; [ABA]_xylem_, **E–H**) in AC and *flacca* grown under ambient (400 ppm) and elevated (800 ppm) CO_2_ concentrations during progressive soil drying. CON indicates the control treatment sprayed with water, +ABA indicates exogenous ABA application. FTSW indicates the fraction of transpiration soil water. *, **, and *** indicate the significant differences between ABA treatments by Student’s *t*-test at *p* < 0.05, *p* < 0.01, *p* < 0.001, respectively; ns denotes no significant difference. Error bars indicate the standard error of the means (S.E.) (*N* = 3–4).

*The data is presented in Figures 4, 5.*

### Leaf, Xylem Sap Abscisic Acid Concentrations and Their Relationships With the Fraction of Transpirable Soil Water Threshold

In total, 1 day after exogenous ABA application, the ABA-treated AC plants possessed higher leaf ABA concentration [ABA]_leaf_ than the non-ABA-treated plants, while the ABA-treated *flacca* had both higher [ABA]_leaf_ and xylem sap ABA concentration ([ABA]_xylem_) compared with the non-ABA-treated controls. During soil drying, when FTSW = 0.50, there were interactions of [ABA] and [CO_2_] on [ABA]_leaf_ of both genotypes, which was relatively higher in the ABA-treated plans grown under *e*[CO_2_] than under *a*[CO_2_]. Moreover, at the end of soil drying (i.e., FTSW = 0.05), significantly higher [ABA]_xylem_ was observed in the ABA-treated *flacca* than the non-ABA-treated *flacca* plants, accompanied by an interaction of [CO_2_] and [ABA]. Drought stress increased both [ABA]_leaf_ and [ABA]_xylem_ at the end of soil drying though being less significant in *flacca* (Figure 5 and Table 3).

**FIGURE 6:**
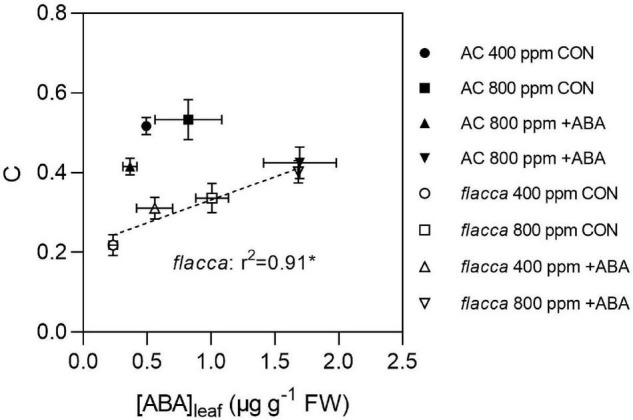
Relationship between the FTSW threshold at which *g*_s_ started to decline (C) and the leaf ABA concentrations ([ABA]_leaf_) at second harvest before FTSW reaching C in AC and *flacca* grown under ambient (400 ppm) and elevated (800 ppm) CO_2_ concentrations. CON indicates the control treatment sprayed with water, +ABA indicates exogenous ABA application. * Indicate significant difference of the regression line of *flacca* at *p* < 0.05. Error bars indicate the standard error of the means (S.E.) (*N* = 3–4).

The relationships of the FTSW threshold at which *g*_s_ started to decline (C) and [ABA]_leaf_ at the second harvest (FTSW value was close to C) in AC and *flacca* are shown in Figure 6. The results showed that only in *flacca*, C was linearly correlated with [ABA]_leaf_, whereas C of AC did not respond to increasing [ABA]_leaf_. Moreover, no obvious relationship between C and [ABA]_xylem_ was observed in either genotype.

### Principle Component Analysis Plot of *g*_s_, Water Relations, Leaf, and Xylem Abscisic Acid Concentrations of the Well-Watered Plants

**FIGURE 7:**
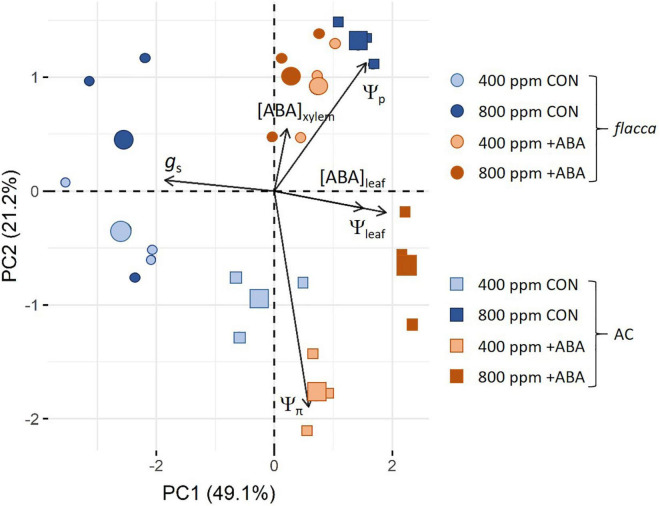
Principal component analysis of stomatal conductance (*g*_*s*_), plant water relations, including leaf water potential (Ψ_leaf_), osmotic potential (Ψ_π_), and turgor pressure (Ψ_p_), and leaf and xylem sap ABA concentrations ([ABA]_leaf_, [ABA]_xylem_) in well-water AC and *flacca* grown under ambient (400 ppm) and elevated (800 ppm) CO_2_ at the time of the first harvest. CON indicates the control treatment sprayed with water, +ABA indicates exogenous ABA application. The contributions of each PCA axis (PC1 and PC2) are indicated on the graph.

Principle component analysis plot of *g*_s_, water relations, leaf, and xylem ABA concentrations of well-watered AC and *flacca* is depicted in Figure 7. PC1 and PC2 axes explained 70.3% of cluster formation, with 49.1% attributed to PC1 and 21.2% to PC2. Overall, AC under all treatments and ABA-treated *flacca* were clustered toward the right side of the PCA plot, whereas all non-ABA-treated *flacca* were clustered to the left in the same direction as the *g*_s_ vector, indicating non-ABA-treated *flacca* had higher *g*_s_ than other plants. The clustering of the [AC, 800 ppm, +ABA] indicated a covariation between Ψ_leaf_ and [ABA]_leaf_ under this treatment, which was negatively associated with *g*_s_. [ABA, *flacca*] and [AC, 800 ppm, CON] treatments were grouped along with PC2, showing a close correlation with [ABA]_xylem_ and Ψ_p_, and no correlation with *g*_s_. Furthermore, [AC, 400 ppm] was opposed by Ψ_p_ and [ABA]_xylem_, being clustered to the bottom of the plot in the same direction as Ψ_π_ vector.

### Relationships Between Stomatal Conductance, Leaf Abscisic Acid Concentration, and Leaf Turgor

**FIGURE 8:**
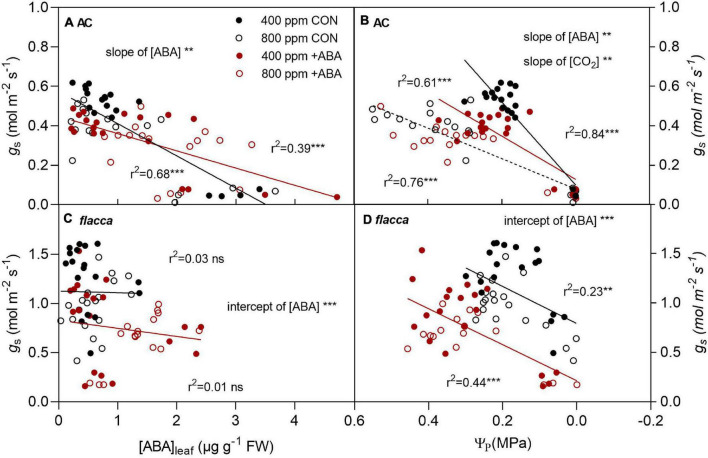
Relationships between stomatal conductance (*g*_s_) and leaf ABA concentration ([ABA]_leaf_), *g*_s_ and turgor pressure (Ψ_p_) in AC **(A,B)** and *flacca* **(C,D)** grown under ambient (400 ppm) and elevated (800 ppm) CO_2_ during soil drying. CON indicates the control treatment sprayed with water, +ABA indicates exogenous ABA application. ** and *** indicate significant differences in the regression lines and significant differences of the slopes and intercepts between treatments at *p* < 0.01 and *p* < 0.001, respectively; ns denotes no significant difference. Black and red lines (denote CON and +ABA, respectively) indicated that there were significant differences between [ABA] treatments. Solid and dotted black lines in panel **(B)** indicated that only under CON but not +ABA there was a significant difference between [CO_2_] treatments.

In AC, there were linear relationships between *g*_s_ and [ABA]_leaf_, *g*_s_ and Ψ_π_, where *g*_s_ declined linearly with increasing [ABA]_leaf_ and decreasing Ψ_π_ (Figure 8). Exogenous ABA application decreased the slope of the regression line of *g*_s_ and [ABA]_leaf_, while *e*[CO_2_] had no influence on it. Regarding the regression line of *g*_s_ and Ψ_π_, the slopes differed between ABA-treated and non-ABA-treated plants, whereas *e*[CO_2_] only decreased the slope in non-ABA-treated plants. In *flacca*, there was no significant relationship between *g*_s_ and [ABA]_leaf_ under either treatment, while g_s_ decreased linearly with declining Ψ_π_. In addition, the intercepts of these regression lines differed between [ABA] treatments due to the initial low levels of g_s_.

## Discussion

Plant hormone ABA is a vital factor in both *e*[CO_2_]- and the drought stress-mediated plant water relation regulatory network, and ABA homeostasis is essential for plants to cope with drought stress. In the present study, we investigated different effects of exogenous ABA application and *e*[CO_2_] as well as their interactions on tomato leaf gas exchange and water relation characteristics during progressive soil drying.

### Plant Growth and Stomatal Conductance as Influenced by Elevated CO_2_ and Exogenous Abscisic Acid

It is well known that *e*[CO_2_] decreases *g*_s_ while stimulating carbon assimilation, thus improving plant drought tolerance (Li S. et al., 2020). In tomato plants, previous studies have shown that the plant response to *e*[CO_2_] was attenuated or absent in ABA-deficient mutant *flacca*, affirming the obligatory role of ABA in CO_2_ signaling pathway (Fang et al., 2019; Wei et al., 2020). Consistent with this, here LDW and SDW of AC plants were improved by *e*[CO_2_] during progressive soil drying, but these stimulations were less significant in *flacca*. Upon exogenous ABA application, retarded plant growth of *flacca* was rescued under both watering conditions, while growth of AC plants did not benefit from exogenous ABA treatment (Table 1), which was consistent with previous study on tomato and confirmed the important role of ABA in maintaining the shoot development (Sharp et al., 2000; Aroca et al., 2008).

It has been reported that application of exogenous ABA inhibited water loss and reduced the rate of soil drying due to stomatal closure (Hossain et al., 2015; He et al., 2019). In the present study, in both ABA-treated AC and *flacca*, slower soil water depletion during progressive soil drying was recorded, which coincided with the decreases of *SA* and *g*_s_ in relation to the non-ABA-treated controls (Figures 1–3). However, in the drought-stressed AC plants, only those grown under *e*[CO_2_] possessed slower rate of soil water depletion upon exogenous ABA application compared with the non ABA-treated plants, indicating a significant interaction of *e*[CO_2_] and exogenous ABA on plant water consumption when soil water was limited. Although in AC *e*[CO_2_] accelerated water consumption due to stimulated plant growth, exogenous ABA application had no influence on plant growth (Figure 1 and Table 1). Therefore, the interactive effect of *e*[CO_2_] and exogenous ABA on water consumption rate might be ascribed to the altered stomatal behavior, as both factors could induce stomatal closure.

It is worth noting that upon exogenous ABA treatment the depressive effect of *e*[CO_2_] on *SA* was eliminated in both genotypes (Figure 2). Considering that *e*[CO_2_] had exerted inhibiting effects on stomatal aperture, it could be assumed that exogenous ABA application overrode the effect of *e*[CO_2_] on stomatal movement. However, the common view is that ABA can amplify the effects of [CO_2_] on stomatal behavior (Engineer et al., 2016), and *e*[CO_2_]-grown soybean showed a stronger response to endogenous ABA (Gray et al., 2016), though ABA and [CO_2_] signal transductions also could be independent (Hsu et al., 2018). As our previous studies demonstrated that severe drought stress overrode the *e*[CO_2_] effects, here exogenous ABA application exhibited the same influences as drought stress. Furthermore, decreases in *SA* and *g*_s_ were more pronounced in *flacca* than AC (Figures 2, 3). Namely, the stomata of *flacca* exhibited greater sensitivity to exogenous ABA than AC due to ABA deficit. Interestingly, after the onset of soil drying, before the occurrence of stomatal closure, there were significant increasing trends of *g*_s_ in the ABA-treated *flacca* while the ABA-treated AC possessed a constantly lower *g*_s_ before the FTSW thresholds (Figure 3), indicating that the effects of exogenous ABA application lasted for a longer period in AC than in *flacca*. Early studies have found that the abnormal stomatal behavior in *flacca* was associated with the high level of ethylene resulting from an ABA deficit (Tal et al., 1979), and other chemical signals could also counteract the ABA signaling, as is the case with cytokinin (Prerostova et al., 2018), which might accelerate the degradation process of exogenous ABA in *flacca* plants. In addition, *flacca* is known to be impaired in the oxidation of ABA aldehyde to ABA (Sagi et al., 2002), vanishing effects of exogenous ABA might be ascribed to the redistribution of ABA to other plant tissues. These results suggest the necessity to explore the regulation of ABA homeostasis in the two genotypes in future studies. Despite the significantly lower g_s ini_ in ABA-treated *flacca*, the *g*_s end_ of *flacca* was still higher than AC under all treatments (Figure 3 and Table 2), which indicated that *flacca* could not fully close stomata under severe drought stress after exogenous ABA application. In another ABA-deficient mutant *sitiens*, a decrease in the leaf gas exchange rate by exogenous ABA (100 μM) was significant after 52 days of mild drought stress, but it still possessed a higher level of transpiration rate than wild type (Aroca et al., 2008). Therefore, exogenous ABA priming before soil drying could not sufficiently induce stomatal closure under severe drought stress.

Previous studies on tomato revealed that *e*[CO_2_] retarded stomatal closure during soil drying due to reduced stomatal sensitivity to ABA (Liu et al., 2019; Wei et al., 2020). However, here we did not find *e*[CO_2_] delayed stomatal closure in AC, the reason behind this discrepancy is unknown, which might be ascribed to the varied experimental conditions between the different studies. By contrast, in *flacca* plants, exogenous ABA application sensitized the stomatal response to drought though only significant under *a*[CO_2_] (Figures 3C,D and Table 2), suggesting that the stomatal closure in ABA-deficient mutant could be advanced by exogenous ABA treatment, which might contribute to water-saving under drought stress. In addition, *e*[CO_2_] decreased *g*_s_ in *flacca* despite the slight influence on *SA*, and these effects were absent in ABA-treated *flacca*. Although the retarded stomatal response to *e*[CO_2_] in *flacca* was attributed to ABA deficit (Wei et al., 2020; Li et al., 2021), exogenous ABA application could not recover the response due to its strongly induced stomatal closure.

### Plant Water Relations as Influenced by Elevated CO_2_ and Exogenous Abscisic Acid

Previous studies have suggested that exogenous ABA application is beneficial for plants to improve drought tolerance as a result of induced stomatal closure, enhanced water relations, and a higher OA (Du et al., 2013; He et al., 2019). In the present study, 1 day after exogenous ABA application, higher Ψ_leaf_, Ψ_π_, and Ψ_p_ were observed compared to the non-ABA-treated plants (Figure 4), confirming the positive effects of exogenous ABA on plant water relations. In an early study with exogenous ABA application (10 μM) on *flacca*, the authors found that the impaired shoot growth could be restored but no improved Ψ_leaf_ was observed (Sharp et al., 2000). As stimulation on expansive growth by ABA is primarily related to hydraulic control (Tardieu et al., 2015), here the improved water status in ABA-treated *flacca* might be responsible for the restored growth (Table 1). Moreover, the positive effects of exogenous ABA on plant water relations were more pronounced in *flacca* than in AC (Figure 4), coinciding with the stomatal responses in the two genotypes. In wild-type tomato and ABA-deficient mutant *sitiens*, Aroca et al. (2008) found that the genotypes showed different gene regulatory patterns in response to exogenous ABA, including ABA biosynthesis-related and aquaporin-encoding genes, which might be associated with the high sensitivity to ABA in *flacca* plants. Surprisingly, at the end of soil drying, the improved water relations, by application of exogenous ABA, vanished in both genotypes, including Ψ_p_ (Figure 4 and Table 3). Thereby, the decreased *g*_s_ in ABA-treated *flacca* at the end of soil drying could not be explained by modulated water relations. In addition, OA is also considered as a vital trait for maintaining leaf turgor, and increased OA accompanied by improved leaf water relations have also been reported in exogenous ABA-treated soybean (He et al., 2019). However, here we did not find the influence of exogenous ABA on OA in both genotypes during the whole period of soil drying. Therefore, more detailed mechanisms related to the ABA-modulated plant water relations merit future studies.

The positive effects of *e*[CO_2_] on plant growth and physiology are reported to be more pronounced under drought stress which, however, depends on the severity of drought stress (Leakey et al., 2006; Gray et al., 2016). In the present study, the well-watered AC plants grown at *e*[CO_2_] had an improved Ψ_leaf_, Ψ_π_, and Ψ_p_ in relation to those grown under *a*[CO_2_], but these effects were eliminated during soil drying (Figure 4 and Table 3), in line with previous findings (Wei et al., 2020). As *e*[CO_2_]-grown plant already possessed improved water relations, effects of exogenous ABA on AC became less significant at *e*[CO_2_]. In Figure 8, *e*[CO_2_] rendered a less sensitivity of stomata to Ψ_π_, but this effect was absent in ABA-treated AC. Collectively, these results implied an offsetting effect of [CO_2_] and exogenous ABA on plant water relations as well as stomatal behavior.

### Abscisic Acid Signal Mediates Stomatal Drought Response

In the present study, 1 day after exogenous ABA application, significant increases of [ABA]_leaf_ in AC and *flacca* were observed, and the increase was more pronounced in *flacca* than in AC, though it was gradually diminished during soil drying (Figure 5 and Table 3). As oxidative degradation of ABA could occur rapidly (Zhang et al., 1995), in tomato plants, when ABA-treated plants were exposed to long-term drought (52 days), ABA contents in plants did not differ between the ABA-treated and non-ABA-treated plants despite the accumulated exogenous ABA effects on water relations still existing (Aroca et al., 2008). By contrast, here, we found that the vanishing effect on water relations was accompanied by decreased accumulation of [ABA]_leaf_ in ABA-treated *flacca*. More interestingly, the interactions of [CO_2_] and [ABA] on [ABA]_leaf_ of AC and *flacca* indicated that the ABA degradation process might be disturbed under *e*[CO_2_], which could explain the reduced ABA sensitivity in the drought-stressed tomato plants gown under *e*[CO_2_] (Yan et al., 2017; Wei et al., 2020). In ABA-treated *flacca*, an increase in [ABA]_xylem_ was observed especially at the end of soil drying, which was accompanied by an interaction of [CO_2_] and exogenous ABA (Figure 5 and Table 3), implying that *e*[CO_2_] exerted an effect on ABA homeostasis. It is well documented that ABA metabolism, including biosynthesis and degradation, ABA recirculation, and exudation processes are all involved in the regulation of ABA homeostasis (Hartung et al., 2005). Moreover, redistribution of ABA within plant organs has often been reported, and translocation of ABA from leaves to roots can be intensified when roots are experiencing drought (Hartung et al., 2005; Ikegami et al., 2009; Ernst et al., 2010). Taking together, except for the ABA degradation pathway, decrease of [ABA]_leaf_ in the ABA-treated *flacca* could also be ascribed to the redistribution of ABA between shoot and root considering its inability of ABA synthesis in roots.

It is well known that tomato plants are likely to exhibit isohydric behaviors (Moshelion et al., 2015), while ABA-deficient mutant *flacca* and *sitiens* could be transpired more for longer periods during soil drying than AC (Aroca et al., 2008; Wei et al., 2020), representing a near-anisohydric characteristic. Furthermore, rapid ABA biosynthesis might facilitate isohydric behavior (Moshelion et al., 2015). In the present study, before the occurrence of stomatal closure (i.e., when FTSW = 0.50), ABA-treated *flacca* grown at *e*[CO_2_] possessed relatively high [ABA]_leaf_ (Figure 5), which was found to be closely correlated with the FTSW threshold at which *g*_s_ started to decrease (C) (Figure 6). These results raise the possibility that anisohydric behaviors in the ABA-deficient mutant could be shifted toward near-isohydric behaviors through the combined effects of *e*[CO_2_] and exogenous ABA, representing a restored stomatal drought response. The change in the water management strategies has been reported to be activated by different soil water statuses (Zhang et al., 2012), also the overexpression of a key tonoplast aquaporin (Sade et al., 2009), which could be linked to ABA.

Previous studies have shown that shoot-to-root ABA transport played a role in the regulation of water flux and induction of stress-resistant genes in roots (Ikegami et al., 2009). In the near-anisohydric grapevine cultivar Syrah, the authors found that it showed relatively high catabolism of ABA in xylem sap, which might lower its hypersensitivity to water stress (Dayer et al., 2020). Therefore, it could be assumed that reloading of ABA into xylem sap in *flacca* plants might stimulate some specific metabolic process, causing feedback on stomatal movements, which might explain the low *g*_s_ in ABA-treated plants at the end of soil drying but without [ABA]_leaf_ accumulation. For example, early studies on *flacca* have shown that low ABA content stimulated ethylene production, which could be restored to normal levels with exogenous ABA (Tal et al., 1979; Sharp et al., 2000). As ethylene production is often increased by drought stress, it might be associated with the restored drought response in ABA-treated *flacca* as noticed in the present study. In addition, exogenous ABA-induced other metabolic adaption has been reported widely, including stimulated resistant protein patterns (Zhou et al., 2014), enhanced energy storages, and activities of antioxidant enzymes (Latif, 2014; Gai et al., 2020), and these possibilities merit further studies.

Under well-watered conditions, the ABA-treated *flacca* plants possessed the same leaf characteristics as AC in the global PCA plots (Figure 7). However, despite the restored leaf gas exchange rates, water relation characteristics, and ABA concentrations in *flacca* (Figures 3–5), the PCA plot indicated that, under well-watered conditions, exogenous ABA could still not induce a correlation between *g*_s_ and [ABA]_leaf_ in *flacca*, but showed an interaction with Ψ_p_ and [ABA]_xylem_ (Figure 7). Furthermore, during the whole period of soil drying, *g*_s_ was still not correlated with [ABA]_leaf_ but only responded to Ψ_p_ (Figure 8). Therefore, a question arises as to how exogenous ABA application affected *flacca*’s stomatal movements considering the no involvement of [ABA]_leaf_. Pantin et al. (2013) demonstrated that ABA can induce stomatal closure through an indirect hydraulic effect on water permeability within leaf vascular tissues. In the exogenous ABA-fed near-anisohydric grapevine cultivar, the existence of indirect ABA effects on *g*_s_ has also been reported, which was associated with the ABA catabolism (Dayer et al., 2020). Therefore, it could be assumed that the exogenous ABA decreased *g*_s_ in *flacca* plants through an indirect hydraulic effect. Taking together, future research should focus on exploring the correlation of ABA metabolic process and ABA indirect hydraulic effects on stomatal behavior.

## Conclusion

Exogenous ABA application decreased *SA* and *g*_s_, improved plant water relations, including Ψ_leaf_, Ψ_π_, and Ψ_p_, and increased [ABA]_leaf_ in both AC and *flacca*, though these two genotypes showed differential responses during soil drying, where exogenous ABA priming sensitized the *g*_s_ response to soil drying in *flacca*. In both ABA-treated genotypes, high [ABA]_leaf_ lasted for a longer period under *e*[CO_2_] than *a*[CO_2_] conditions, which might be associated with ABA degradation or redistribution and responsible for the *e*[CO_2_]-induced ABA insensitivity. In AC, depression on *SA* and improvement in water relations by exogenous ABA was more pronounced in *a*[CO_2_]-grown plants compared to their *e*[CO_2_]-grown counterparts, and *e*[CO_2_] only lowers *g*_s_ sensitivity to Ψ_p_ in non-ABA-treated AC, indicating that *e*[CO_2_] could counteract the effects of exogenous ABA. In *flacca*, the effects of exogenous ABA on *g*_s_ were gradually diminished during soil drying. However, ABA-treated *flacca* still showed a partly restored stomatal drought response at both [CO_2_] conditions, and was accompanied by the recovered plant growth and increased [ABA]_xylem_ especially under *e*[CO_2_]. Thus, the restored drought response in the absence of accumulation of [ABA]_leaf_ could be associated with ABA-stimulated metabolic adaptions. Although ABA-treated *flacca* exhibited recovered stomatal behavior, the PCA plot and regression analysis showed that [ABA]_leaf_ was not responsible for the decreasing *g*_s_. Therefore, our results raise the possibility that exogenous ABA-induced stomatal closure in *flacca* could be attributed to an indirect hydraulic effect.

## Data Availability Statement

The original contributions presented in the study are included in the article/Supplementary Material, further inquiries can be directed to the corresponding author/s.

## Author Contributions

Both authors conceived the concept, carried out the experiment, and have read and agreed to the published version of the manuscript. SL wrote the manuscript with support from FL. FL supervised the project.

## Conflict of Interest

The authors declare that the research was conducted in the absence of any commercial or financial relationships that could be construed as a potential conflict of interest.

## Publisher’s Note

All claims expressed in this article are solely those of the authors and do not necessarily represent those of their affiliated organizations, or those of the publisher, the editors and the reviewers. Any product that may be evaluated in this article, or claim that may be made by its manufacturer, is not guaranteed or endorsed by the publisher.

## Supplementary Material

Supplementary Figure 1 The average temperature, relative humidity, vapor pressure deficit (VPD), and CO_2_ concentration [CO_2_] in 400 and 800 ppm greenhouse cells of AC and *flacca* during the whole experimental period.
