# Supplementary figures and images for: Exogenous Abscisic Acid Priming Modulates Water Relation Responses of Two Tomato Genotypes With Contrasting Endogenous Abscisic Acid Levels to Progressive Soil Drying Under Elevated CO2
Source: Front Plant Sci. 2021 Nov 24;12:733658. doi: 10.3389/fpls.2021.733658 (PMC8651563; doi:10.3389/fpls.2021.733658)

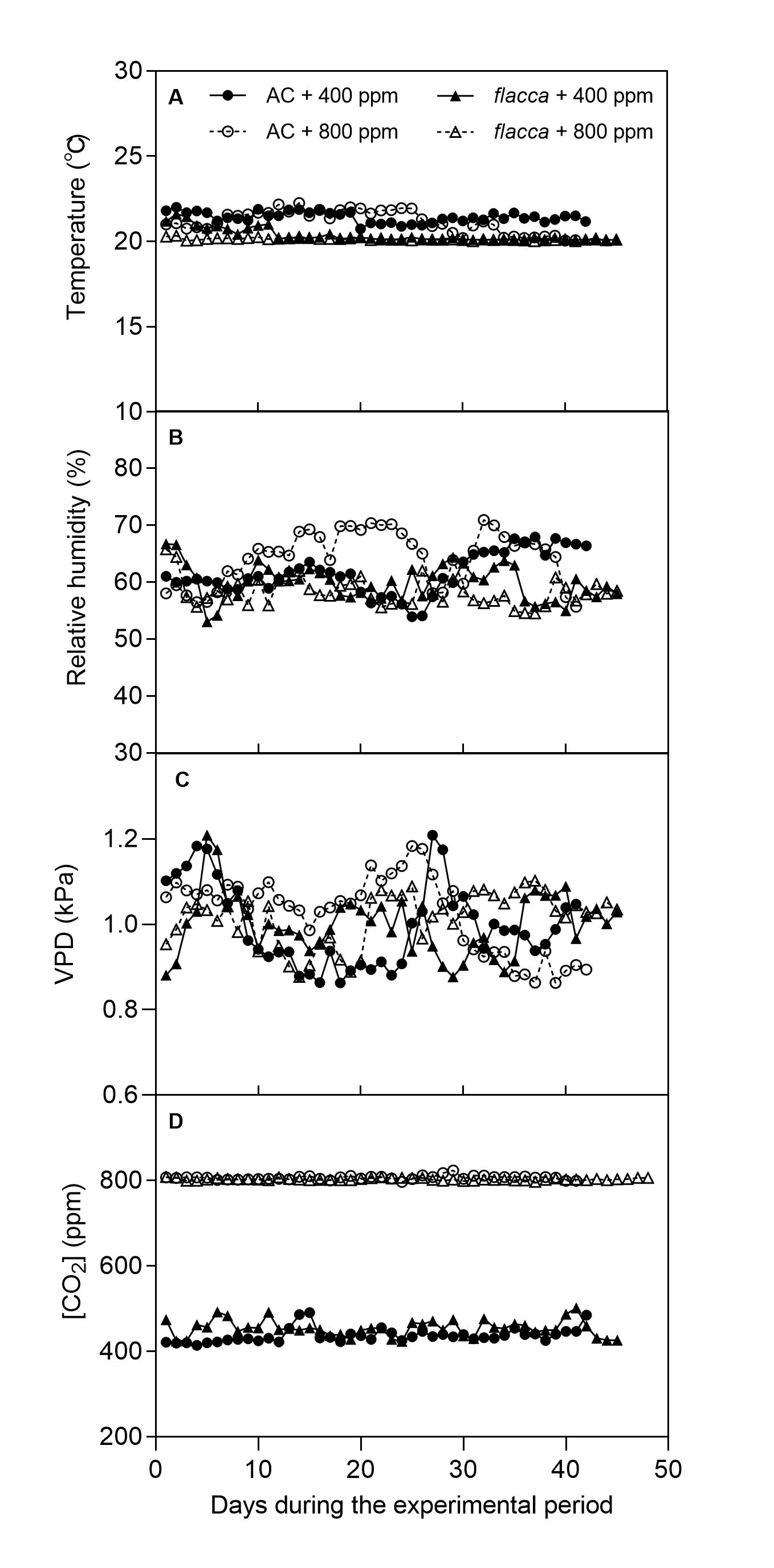

Supplement: Supplementary Figure 1 — The average temperature, relative humidity, vapor pressure deficit (VPD), and CO2 concentration [CO2] in 400 and 800 ppm greenhouse cells of AC and flacca during the whole experimental period. [file Image_1.jpg]
